# Getting trapped in a dead end? Trait self-control and boredom are linked to goal adjustment

**DOI:** 10.1007/s11031-022-09943-4

**Published:** 2022-06-15

**Authors:** Maik Bieleke, Wanja Wolff, Lucas Keller

**Affiliations:** 1 Department of Sport Science, University of Konstanz, 78457 Konstanz, Germany; 2 Department of Educational Psychology, University of Bern, 3012 Bern, Switzerland; 3 Department of Psychology, University of Konstanz, 78457 Konstanz, Germany

**Keywords:** Goal adjustment, Self-control, If–then planning (implementation intentions), Boredom, Boredom proneness

## Abstract

Disengaging from unattainable goals and reengaging in alternative goals is essential for effective goal pursuit; yet, surprisingly little is known about associated personality factors. Here, we focused on individual differences in self-control (domain-general self-control, if–then planning) and boredom (boredom proneness, boredom avoidance and escape tendencies). Concerning goal adjustment in everyday life (Study 1; *N* = 323 crowdworkers), if–then planning was associated with worse disengagement and better reengagement. While boredom proneness was associated with poorer reengagement, boredom avoidance and escape tendencies were associated with better reengagement. When goal striving was thwarted during the COVID-19 pandemic (Study 2; *N* = 97 students), similar associations emerged along with links to anxiety and depression. However, disengagement was no longer associated with if–then planning but instead with better self-control and higher boredom proneness. These results show differential relationships of goal disengagement and reengagement with self-control and boredom, paving the way to a better understanding of who struggles or shines when effective goal adjustment is required.

## Introduction

Failing to accomplish a goal is a cumbersome experience people make more often than they would like. This failure can have many reasons: a realization that one’s skills, resources, and capabilities are insufficient, a fleeting opportunity or an unexpected obstacle, or changing circumstances like illness or job loss that prevent goal attainment. During the COVID-19 pandemic, for instance, people around the globe were suddenly forced into a situation that prevented them from attaining their goals (Ritchie et al., 2020). When recognizing such a failure, people may need to adjust their goal striving if the goal turns out to be futile. Successful goal adjustment involves two main steps (e.g., Wrosch et al., 2003): First, it requires to disengage from the futile goal, cease goal-directed activities, and reduce mental and emotional involvement. Second, goal adjustment also involves to get back on track, reengage in new meaningful pursuits, or put effort toward reaching concurrent goals that are still feasible (and desirable). It is well-known that people differ in how effectively they master these challenges, with various downstream consequences for their mental health and well-being (review by Wrosch & Scheier, 2020). In a recent meta-analysis of more than 30 independent samples, Barlow et al. (2020) showed that effective goal disengagement and reengagement are linked to better psychological well-being and a higher quality of life. Unfortunately, little is known about the personality factors related to differences in goal disengagement and reengagement, thereby limiting the knowledge about possible antecedents of effective goal adjustment (Wrosch & Scheier, 2020; Brandstätter & Bernecker, 2022). Here, we adopt an individual differences approach to address this question and investigate factors that might hinder or help people to effectively adjust their goal striving. While there likely is a large number of personality factors involved in the decision to adjust one’s goals, we took a first step by focusing on self-control and boredom. These two constructs are thought to assume complementary functions in steering behavior toward goal adjustment or away from it (e.g., Wolff & Martarelli, 2020; Bieleke & Wolff, 2021) and therefore provide a promising and parsimonious starting point.

## Self-control and the self-control strategy of if–then planning

Self-control refers to the “efforts people exert to stimulate desirable responses and inhibit undesirable responses” (de Ridder et al., 2012, p. 77), giving it a vital function for attaining valued goals (Baumeister et al., 2007; Duckworth & Kern, 2011). For instance, in order to have good chances on the job market, students engage in effortful activities that bring them closer to this goal (e.g., attending classes at the university) and refrain from activities that might put them off track (e.g., declining a tempting invitation to party the night before an exam). While self-control is essential to attain challenging goals, it does so by fostering steadfast goal striving (e.g., Ainslie, 2021). Indeed, during the COVID-19 pandemic, individuals with higher self-control were more likely to stick to their already established behaviors and to even adopt novel behaviors to keep pursuing their still valued goals (Kokkoris & Stavrova, 2021). In turn, people with high self-control might find it difficult to adjust their goals to changing circumstances; instead, they might put effort into goal-directed activities even when their chances to bring goals to a successful close are fading. This issue has so far received little attention, and the existing evidence is inconsistent with regard to whether self-control helps or hinders goal adjustment (e.g., Barber et al., 2012; Lee et al., 2018).

People can and do use a wide variety of self-control strategies (e.g., strategies that focus on modifying the situation versus strategies that modulate attentional deployment; Duckworth et al., 2016; Hennecke et al., 2019). These strategies arguably differ in how (in-)flexible they render goal adjustment, and this might explain the inconsistent findings regarding the relationship between self-control and goal adjustment. Accordingly, we focus on one specific self-control strategy that is commonly assumed to render goal striving relatively inflexible: if–then planning (implementation intentions; Gollwitzer, 1999, 2014). When making if–then plans, individuals commit to a certain course of action by mentally linking a critical situation (e.g., an opportunity to act toward the goal) and a goal-directed behavior (e.g., how to seize the opportunity) in an if (situation)-then (behavior) format. If–then planning facilitates goal attainment (reviews by Gollwitzer & Oettingen, 2019; Keller et al., 2020; Bieleke et al., 2021b) by automating the detection of critical situations as well as the initiation of goal-directed behaviors (e.g., Janczyk et al., 2015). However, this automaticity comes at the cost of making goal striving less flexible (Gollwitzer et al., 2008; but note that implementation intentions can be used to increase flexibility in adjusting goal-directed behaviors; Henderson et al., 2007). For instance, individuals might miss good opportunities they did not consider in their plans (Masicampo & Baumeister, 2012), hold on to goal-directed behaviors even when they start to inflict costs (Legrand et al., 2017), or jeopardize their performance in non-planned situations (Bieleke et al., 2018). Importantly, the automation of behavior by if–then planning might impair disengagement from unsuccessful, undesirable, or unfeasible goals as well because it creates “instant habits” (Gollwitzer, 1999) that are hard to break. Additionally, planners may stick to their goal for longer because they are invested in their plans or perceive their goal to be feasible for longer (e.g., Freydefont et al., 2016). Moreover, it is conceivable that some plans will still affect behavior after the goal should have been abandoned or during an action crisis (Brandstätter et al., 2013; Herrmann & Brandstätter, 2015). On the flip side, when it comes to goal reengagement, if–then planning might help get on track early or evoke the feeling of having progressed toward the goal (Gollwitzer et al., 2009). A general tendency to engage in planning as a habitual self-control strategy might further direct more cognitive effort to new goals, increasing their perceived attainability and lowering anticipated task difficulty (e.g., Gendolla et al., 2019). In turn, this commitment right at the beginning of goal pursuit might lead to higher goal reengagement scores. Thus, while planning might render individuals rather inflexible for effective goal disengagement, it might help them reengage in alternative goals more effectively.

There is a long-standing tradition of conceiving self-control as an individual difference variable (Tangney et al., 2004). However, the bulk of research on if–then planning has investigated the effects of instructing participants how to form plans for a goal (e.g., Chapman & Armitage, 2012) or simply assigning participants a suitable if–then plan (e.g., Keller et al., 2021). Accordingly, evidence that if–then planning is detrimental to the flexibility of goal striving rests on studies in which the plans pertained to a single, specific goal. However, there are individual differences in the general inclination to make if–then plans in everyday life (Bieleke & Keller, 2021), and it has not yet been investigated whether this disposition renders goal striving more or less rigid.

## Boredom

Boredom is an aversive experience that occurs when individuals fail to successfully engage in enjoyable activities (Eastwood et al., 2012). Functional accounts of boredom argue that its aversiveness gives boredom an important function in goal-directed behavior (e.g., Bench & Lench, 2013, 2019): it signals that it might be better to let go of the activities pertaining to the current goal and prompts people to engage in activities that serve potentially more rewarding goals instead. In line with this idea, neuroscientific evidence shows that boredom increases the sensitivity for rewards (Milyavskaya et al., 2019b). As a result, the current goal becomes devalued relative to other goals, and the costs associated with its maintenance increase (Bieleke & Wolff, 2021). Boredom should therefore increase the probability that people disengage from their current goal and engage in a new goal (e.g., Bieleke et al., 2021a). Reinforcing this notion, computational and empirical work shows that boredom plays a pivotal role in exploring the environment (Geana et al., 2016; Gomez-Ramirez & Costa, 2017). Therefore, it is plausible that boredom facilitates goal disengagement and reengagement also in response to goals that can no longer be attained.

That being said, it is essential to note that most research on individual differences in boredom focuses on *boredom proneness*, which refers to the “tendency toward experiencing boredom” ( Farmer & Sundberg, 1986, p. 5). People high in boredom proneness experience boredom more often and more frequently; in particular, they tend to perceive their entire life as boring (Tam et al., 2021). This suggests that boredom-prone individuals fail to change their course of action when boredom signals that doing so might be worthwhile. Consequently, while the experience of boredom itself should encourage people to more readily adjust their goal striving, people high in boredom proneness might find it challenging to do so. Corroborating this assumption with regard to both goal disengagement and reengagement, boredom proneness has been aptly characterized as a “failure to launch” (Mugon et al., 2018) and associated with an inclination to procrastinate (Vodanovich & Rupp, 1999).

Moreover, boredom proneness is strongly associated with poor mental health and well-being (e.g., Sommers & Vodanovich, 2000). Interestingly, both current theorizing about boredom (Wolff & Martarelli, 2020; Bieleke & Wolff, 2021) and empirical research (e.g., Bieleke et al., 2021a) suggest strong negative links between self-control and boredom proneness. This indicates, first, that boredom proneness and self-control might have opposing effects on goal adjustment and, second, that it is advisable to control for the respective other construct when examining the individual effects (Wolff & Martarelli, 2020).

While boredom proneness is by far the most common conceptualization of individual differences in boredom (Vodanovich & Watt, 2016), the conceptual overlap between boredom proneness and self-control makes it difficult to examine the relationship between goal adjustment and boredom. Therefore, we additionally turned to individual differences in the tendency to avoid and escape boredom (Bieleke et al., 2021d). The reasoning for such differences is derived from an analysis of the function of pain as a signal that instigates behavior change (Danckert & Eastwood, 2020). Boredom’s proposed core function is to signal that what one is currently doing is not sufficiently rewarding (Eastwood et al., 2012) and that one should search for more rewarding alternatives (Bench & Lench, 2013, 2019). Just as with pain, the signals’ intensity indicates how urgent a change is needed. People differ in their tendency to avoid pain altogether (Nielsen et al., 2009), and in the same vein, individual differences in the tendency to avoid boredom have been proposed (Bieleke et al., 2021d). Importantly, this urge to change is assumed to reflect a very basic sensitivity to the state of the internal or external environment (i.e., how boring it is) that prompts any (adaptive or maladaptive) behavioral response suitable to alter the current state of affairs. In turn, it should not be linked to higher-order processes, such as the decision to employ self-control to follow what has been referred to as boredom’s “call to action” (Danckert, 2020). Moreover, it is plausible that people with a greater urge to respond to boredom are also more likely to readily disengage from futile goals and engage in alternative goals.

## Present research

In two studies, we investigated the relationship between goal adjustment in its two facets, goal disengagement and goal reengagement, and individual differences in self-control and boredom. We assessed goal adjustment as both a general tendency and in response to a specific situation: In Study 1, we asked English-speaking participants sampled from an online crowdsourcing platform about their goal adjustment in general. In Study 2, we asked German-speaking university students sampled from the local subject pool about their goal adjustment during the COVID-19 pandemic. Across both studies, our measures of self-control tapped into general self-control and the self-control strategy of if–then planning, while our measures of boredom tapped into boredom proneness as well as boredom avoidance and escape tendencies.

Although the literature on how self-control and boredom relate to goal adjustment is sparse and largely inconsistent, conceptual considerations allowed us to derive some tentative expectations about the nature of these relationships. Regarding self-control, both positive and negative relations with goal disengagement were plausible given the inconsistent findings in previous research and we investigated this research question an explorative fashion. However, negative relations seemed likely when it comes to if–then planning due to its automating effects on goal striving. This expectation does not hold for goal reengagement, as if–then planning might help people to engage in new goals more effectively. Different predictions arise for the tendency to avoid and escape boredom and boredom proneness. Specifically, theoretical and empirical work makes it plausible to expect that a tendency to avoid and escape boredom is positively associated with goal disengagement and reengagement. In contrast, negative associations were expected for boredom proneness because it taps into the failure to adequately respond to boredom. We examined these research questions in an exploratory fashion and did not preregister the hypotheses.

## Study 1: goal adjustment as general trait

The main aim of Study 1 was to investigate the relationship between goal adjustment (i.e., goal disengagement, goal reengagement), self-control (i.e., domain-general self-control, if–then planning), and boredom (i.e., boredom proneness, boredom avoidance and escape tendencies). Moreover, we examined whether individual differences in self-control and boredom reliably predict individual differences in goal adjustment.

## Method

All materials and data for Study 1 can be found at the OSF (osf.io/yv93q/). Below, we report the variables used in the present research. We also included a scale measuring whether people tend to respond to boredom in adaptive or maladaptive ways for piloting another study; data from this scale were not analyzed in the present research.

### Participants, design, and sample size and power considerations

We recruited 339 participants from Amazon’s MTurk via CloudResearch (Litman et al., 2017) in July of 2021, and 328 completed the study. Three participants failed the attention check at the beginning of the survey, and two participants indicated that they did not answer the questions carefully, meaning that our final sample consists of the remaining 323 participants (137 females, two preferred not to indicate their gender; median year of birth = 1983). Participants received $1.00 for their participation in the 5–6 min survey. We set out to recruit at least 250 participants to reliably test stable correlations (Schönbrodt & Perugini, 2013). The final number of participants allows testing for correlations of *r* ≥ 0.16 with 80% power (Faul et al., 2009).

### Procedure

Participants first gave their informed consent and then performed a quick warm-up task that served as an attention check (Oppenheimer et al., 2009). They then filled out the questionnaires pertaining to goal adjustment, self-control, and boredom in random order before giving their demographics. At last, they could indicate whether they had answered the questions carefully (highlighting that it does not affect their compensation).

### Measures

We used the same seven-point scale ranging from *strongly disagree* to *neither agree nor disagree* (midpoint) to *strongly agree* for all of the following scales.

#### Goal Adjustment Scale (GAS)

This scale (Wrosch et al., 2003) comprises four questions on *goal disengagement* (e.g., “…I stay committed to the goal for a long time; I can’t let it go”) and six questions on *goal reengagement* (e.g., “…I tell myself that I have a number of other goals to draw upon”). Please note that in the GAS, participants indicate how they usually react when they are forced to stop pursuing a goal (i.e., when a goal becomes unattainable); therefore, their answers do not pertain to a specific goal. Answers were coded such that higher values indicate stronger inclinations to disengage from goals and reengage in new goals, respectively. Both subscales’ reliability was good to excellent, with Cronbach’s ɑ = 0.83 and Cronbach’s ɑ = 0.92 for disengagement and reengagement, respectively.

#### Brief Self-Control Scale (BSCS) and If–Then Planning Scale (ITPS)

We used the Brief Self-Control Scale (BSCS; Tangney et al., 2004) and the If–Then Planning Scale (ITPS; Bieleke & Keller, 2021) to assess individual differences in domain-general self-control and if–then planning, respectively. The BSCS consists of 13 statements, gauging participants’ agreement to potential self-descriptions like “I am good at resisting temptation” and “Sometimes I can’t stop myself from doing something, even if I know it is wrong.” The BSCS’s reliability was good, Cronbach’s ɑ = 0.90. In the ITPS, participants answered eight questions regarding different aspects of if–then planning (Bieleke & Keller, 2021), for instance, whether they anticipate potential obstacles and plan how to deal with them. Sample items are “I plan the concrete actions I will take toward my goal” and “I am concerned with what setbacks to expect.” The ITPS’s reliability was also good, Cronbach’s ɑ = 0.82.

#### Short Boredom Proneness Scale (SBPS) and Boredom Avoidance and Escape Scale (BAES)

For the construct of boredom, we used the Short Boredom Proneness Scale (SBPS; Struk et al., 2017), which comprises eight statements that gauge participants’ tendencies to experience boredom. Sample items are “In most situations, it is hard for me to find something to do or see to keep me interested” and “Much of the time, I just sit around doing nothing.” The reliability of the SBPS was excellent, Cronbach’s ɑ = 0.92. To capture individual differences in responses to boredom, we used the Boredom Avoidance and Escape Scale (BAES; Bieleke et al., 2021d), which measures the tendency to avoid boring situations or escape them with four items. Sample items include “I’ll do anything to avoid feeling bored” and “When I feel bored, I must do something about it immediately.” The BAES displayed excellent reliability, Cronbach’s ɑ = 0.95.

## Results

### Descriptive statistics and correlations

Descriptive statistics and bivariate correlations of all variables are presented in Table 1. Goal disengagement and goal reengagement were positively correlated with each other, *r* = 0.32, 95% CI [0.22, 0.41], *t* (321) = 6.04, *p* < 0.001. Goal disengagement was negatively correlated with if–then planning, *r* = − 0.16, 95% CI [− 0.26, − 0.05], *t* (321) = 2.87, *p* = 0.004, in line with the assumption that making if–then plans hampers goal adjustment. However, goal disengagement was neither associated with domain-general self-control nor with boredom, *p*s ≥ 0.19. In contrast, goal reengagement was positively correlated with both domain-general self-control, *r* = 0.26, 95% CI [0.15, 0.36], *t*(321) = 4.77, *p* < 0.001, and if–then planning, *r* = 0.35, 95% CI [0.25, 0.44], *t*(321) = 6.63, *p* < 0.001. Regarding boredom, goal reengagement was negatively correlated with boredom proneness, *r* = − 0.32, 95% CI [− 0.41, − 0.22], *t*(321) = 6.02, *p* < 0.001, but positively correlated with boredom avoidance and escape tendencies, *r* = 0.12, 95% CI [0.01, 0.22], *t*(321) = 2.09, *p* = 0.038. As expected, measures of self-control and boredom were negatively associated with each other, and these correlations were small-to-moderate. Interestingly, the largest correlation emerged between domain-general self-control and boredom proneness, in line with previous research showing substantial overlap between these constructs (e.g., Wolff et al., 2022).

**Table 1:** Means, standard deviations, and correlations of the main variables in Study 1

Variable	*M*	*SD*	1	2	3	4	5
1. Goal disengagement (GAS)	3.76	1.30					
2. Goal reengagement (GAS)	5.13	1.13	.32*** [.22, .41]				
3. Domain-general self-control (BSCS)	4.81	1.13	.07 [− .04, .18]	.26*** [.15, .36]			
4. If–then planning (ITPS)	5.26	0.84	− .16** [− .26, − .05]	.35*** [.25, .44]	.31*** [.21, .40]		
5. Boredom proneness (SBPS)	2.68	1.27	− .03 [− .14, .08]	− .32*** [.25, .44]	− .62*** [.21, .40]	− .29*** [− .39, − .19]	
6. Boredom avoidance and escape (BAES)	3.66	1.63	− .06 [− .16, .05]	.12* [.01, .22]	− .10 [− .20, .01]	.11* [.00, .22]	.21*** [.10, .31]

Values in square brackets represent the 95% confidence interval (CI) of the correlation coefficient

*p < .05. **p < .01. ***p < .001

## Psychometric network analysis

**Fig. 1:**
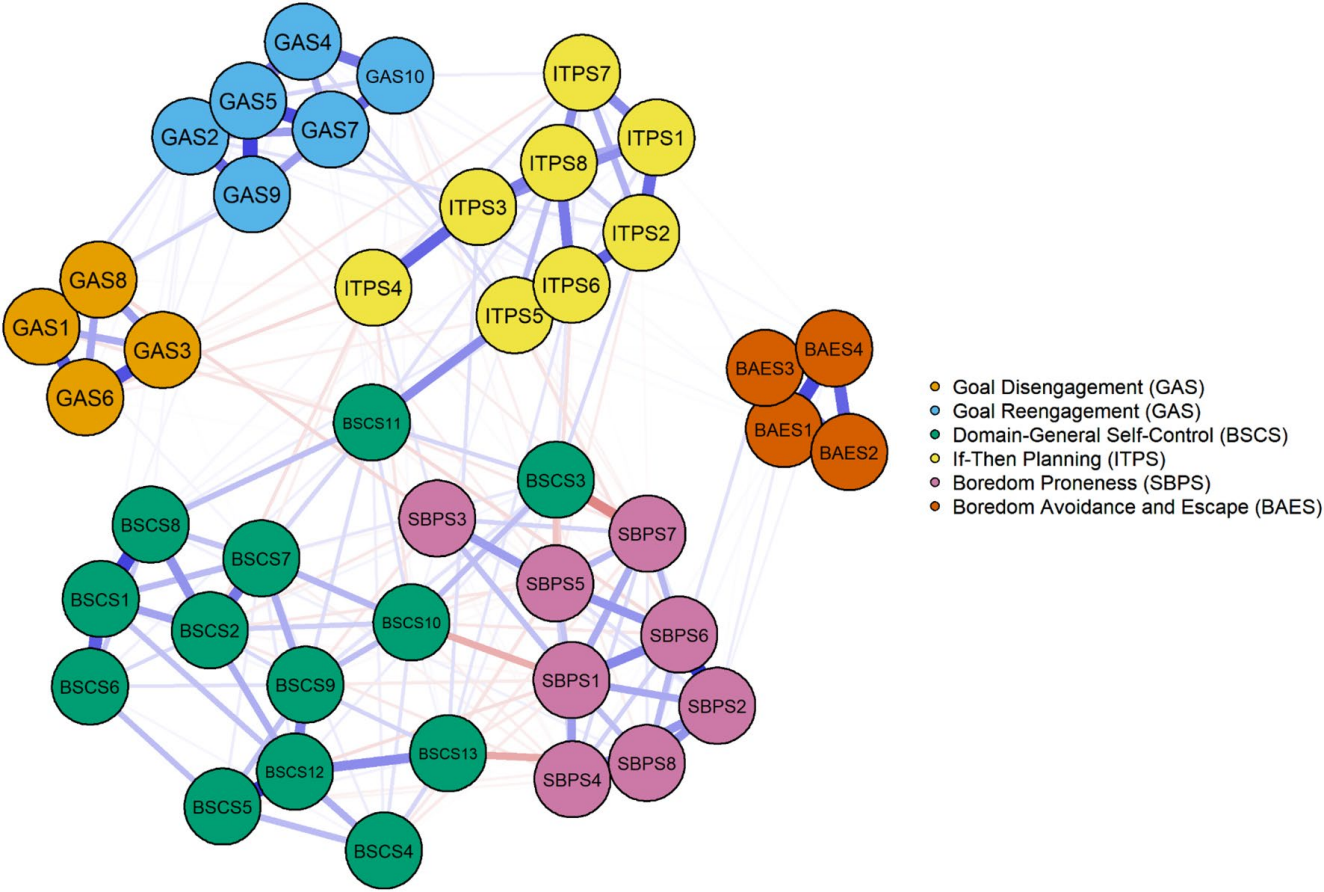
Psychometric network displaying associations between goal adjustment, self-control, and boredom in Study 1. The graph shows individual items as nodes (color-coded by scale) connected by edges. Red and blue edges represent negative and positive associations, respectively, and thicker edges indicate stronger associations. Estimation was based on the Graphical Least Absolute Shrinkage and Selection Operator and the Extended Bayesian Information Criterion to select optimal regularization parameters (EBICglasso)

To better gauge the relationship between goal adjustment, self-control, and boredom beyond bivariate correlations of aggregate scores, we turned to psychometric network analysis (Christensen et al., 2020; Borsboom et al., 2021). This approach allowed us to estimate a parsimonious network of the constructs in which all individual items are represented by nodes. These nodes are linked by edges that represent the regularized partial correlations between them. Accordingly, the network comprises unique associations between nodes that remain after all other nodes in the network have been controlled for. The results of the psychometric network analysis are depicted in Fig. 1. The network has 43 nodes and 241 (out of 903) non-zero edges, suggesting that the structure is indeed parsimonious. The results further suggest goal disengagement and goal reengagement as two distinct communities of GAS items. Also, the nodes representing the GAS items displayed many non-zero edges with nodes representing if–then planning, suggesting a tight association between both constructs. The nodes representing items of the domain-general self-control and boredom proneness scales are closely intertwined, in line with the substantial correlation between both constructs in our study. For instance, item BSCS 3 (“I am lazy”) in Fig. 1 seems more strongly associated with items representing boredom proneness than with items representing self-control. This observation corroborates previous findings that the two constructs are difficult to disentangle with the currently used measures and that the content domains represented by the items tend to overlap (for in-depth discussions, see Bieleke et al., 2021d; Wolff et al., 2022). Taken together, the psychometric network analysis corroborates the observation that if–then planning seems to play a vital role in goal disengagement and goal reengagement.

### Regression analysis

In a final step, we regressed goal disengagement and goal reengagement on our measures of self-control and boredom (Table 2). Both domain-general self-control and if–then planning emerged as significant predictors of goal disengagement. While domain-general self-control was associated with more disengagement, *b* = 0.15, 95% CI [0.02, 0.29], *t*(320) = 2.34, *p* = 0.020; β = 0.13, 95% CI [0.02, 0.25], if–then planning was associated with less disengagement, *b* = − 0.31, 95% CI [− 0.48, − 0.13], *t*(320) = 3.48, *p* < 0.001; β = − 0.20, 95% CI [− 0.31, − 0.09]. However, the effect of domain-general self-control disappeared after adjusting for boredom proneness and boredom avoidance and escape tendencies, which had no effects on goal disengagement themselves, *p*s ≥ 0.35.

**Table 2:** Regressing goal adjustment on self-control and boredom

	Goal disengagement			Goal reengagement		
*N* = 323	M1	M2	M3	M1	M2	M3
Intercept	4.63*** (0.48)	3.95*** (0.21)	4.67*** (0.68)	2.24*** (0.40)	5.50*** (0.17)	3.45*** (0.55)
Domain-general self-control	0.15* (0.07)		0.15 (0.08)	0.17** (0.05)		0.04 (0.07)
If–then planning	− 0.31*** (0.09)		− 0.30** (0.09)	0.40*** (0.07)		0.33*** (0.07)
Boredom proneness		− 0.02 (0.06)	0.00 (0.07)		− 0.32*** (0.05)	− 0.22*** (0.06)
Boredom avoidance and escape		− 0.04 (0.05)	− 0.02 (0.05)		0.13*** (0.04)	0.10** (0.04)
R^2^	.04	.00	.04	.15	.14	.19

The table shows unstandardized regression coefficients (*b*) with standard errors in parentheses. *N* denotes the sample size

*p < .05. **p < .01. ***p < .001

Higher goal reengagement was predicted by both higher domain-general self-control, *b* = 0.17, 95% CI [0.06, 0.27], *t*(320) = 3.05, *p* = 0.002; β = 0.17, 95% CI [0.06, 0.27], and higher if–then planning, *b* = 0.40, 95% CI [0.25, 0.54], *t*(320) = 5.45, *p* < 0.001; β = 0.30, 95% CI [0.19, 0.40]. Concerning boredom, boredom proneness predicted less reengagement, *b* = − 0.32, 95% CI [− 0.41, − 0.23], *t*(320) = 6.75, *p* < 0.001; ꞵ = − 0.36, 95% CI [− 0.46, − 0.25], while boredom avoidance and escape predicted more reengagement, *b* = 0.13, 95% CI [0.06, 0.20], *t*(320) = 3.60, *p* < 0.001; β = 0.19, 95% CI [0.09, 0.30]. Again, the effect of domain-general self-control vanished after adjusting for the boredom measures, *b* = 0.04, 95% CI [-0.09, 0.17], *t*(318) = 0.60, *p* = 0.550; β = 0.04, 95% CI [− 0.09, 0.17].

## Discussion

The results of Study 1 were largely consistent with our hypotheses. First, goal disengagement was robustly and negatively associated with if–then planning, whereas goal reengagement was robustly and positively associated with if–then planning. This suggests that if–then planning might indeed hinder goal disengagement but help goal reengagement. However, these findings were not mirrored in the analysis of domain-general self-control, which displayed a weaker (and in the case of disengagement non-significant) association with goal adjustment and failed to robustly predict these constructs as well. Second, goal reengagement displayed robust negative associations with boredom proneness and robust positive associations with boredom avoidance and escape tendencies. However, there were no associations between goal disengagement and trait measures of boredom. Taken together, these findings highlight a unique and important role of if–then planning for goal adjustment that does not seem to reflect differences in domain-general self-control. Moreover, the results stress the importance of distinguishing between disengagement and reengagement as complementary aspects of goal adjustment that are differentially related to self-control and boredom.

## Study 2: adjusting goals in specific situations

The aims for Study 2 were threefold. First, we examined whether the results of Study 1 apply to specific personal goals that become unattainable due to external factors. The COVID-19 pandemic provides several prototypical examples for such a situation because it has led to various restrictions in daily life, from limiting public gatherings, to the introduction of contact restrictions, to a sudden shift to remote learning for university students. Our focus is on how university students adjusted their goal striving when they realized that their academic or non-academic goals were thwarted by the pandemic. In general, we expected the nature of the associations between goal adjustment, self-control, and boredom to be similar to those observed in Study 1. There is one exception, however, that provides the grounds for the second aim of Study 2: Research on if–then planning suggests that people find it easier to disengage from their plans when goal attainment is unequivocally pointless (e.g., when substantial financial losses loom; Legrand et al., 2017). As the COVID-19 pandemic frustrated many goals in a way that unequivocally discouraged their attainment (e.g., making a semester abroad impossible), we expected that the association between goal disengagement and if–then planning would be reduced. The third and final aim of Study 2 pertained to the downstream consequences of goal adjustment, self-control, and boredom for anxiety and depression symptoms during the pandemic (Löwe et al., 2010). Barlow and colleagues (2020) showed that both goal disengagement and especially goal reengagement were negatively related to anxiety and depression. We additionally expected anxiety and depression to be correlated negatively with domain-general self-control (e.g., Tangney et al., 2004) and positively with boredom (e.g., Sommers & Vodanovich, 2000).

## Method

All materials and data for Study 2 can be found at the OSF (osf.io/yv93q/). Below, we report the variables used in the present research.

### Participants, design, and sample size and power considerations

A total of 111 participants from a German university’s local subject pool completed the study in August and September of 2021. Fourteen of those participants failed the attention check at the beginning of the survey, meaning that our final sample consists of the remaining 97 participants (82 females; median year of birth = 1998). Participants chose between receiving partial course credit or participating in a lottery with a 10% chance of winning €25 (~ $29) for their participation in the 10-min survey. We set out to recruit as many participants as possible in the limited subject pool. The final number of participants allows testing for correlations of *r* ≥ 0.28 with 80% power (Faul et al., 2009).

### Procedure

The procedure was similar to Study 1 except for the following two changes. First, while all of the previously used questionnaires were again presented in random order, the goal adjustment questionnaire always came last and was presented before the demographic questions. We made this change because the goal adjustment questionnaire required a different framing than the other questionnaires due to the focus on a specific goal (see below). Second, participants answered a set of questions pertaining to symptoms of anxiety and depression during COVID-19. We included these variables because anxiety and depression are established downstream consequences of goal adjustment and have also been associated with poor self-control and boredom proneness. As in Study 1, participants indicated whether they had answered the questions carefully at the end of the survey. All participants indicated to have done so.

### Measures

For the questionnaires already used in Study 1, we used the same seven-point scale ranging from *strongly disagree* to *neither agree nor disagree* (midpoint) to *strongly agree* for all of the following scales.

#### Goal Adjustment Scale (GAS)

In Study 2, we used a German translation of the scale (Haase & Wrosch, 2020) and altered the introduction. Instead of asking how participants generally respond when they have to abandon a goal, we asked them for an important goal that they had to abandon due to the COVID-19 pandemic and the associated restrictions on public and social life. Reliabilities of the subscales were acceptable, Cronbach’s ɑ = 0.78, and good, Cronbach’s ɑ = 0.88, for disengagement and reengagement, respectively.

#### Brief Self-Control Scale (BSCS) and If–Then Planning Scale (ITPS)

We used German translations of the BSCS (Bertrams & Dickhäuser, 2009) and the ITPS (Bieleke & Keller, 2021). Reliabilities were good, Cronbach’s ɑ = 0.89, and adequate, Cronbach’s ɑ = 0.75, for the BSCS and the ITPS, respectively.

#### Short Boredom Proneness Scale (SBPS) and Boredom Avoidance and Escape Scale (BAES)

Reliability of the SBPS (in its German version; Martarelli et al., 2021) was good, Cronbach’s ɑ = 0.89, also for the BAES, Cronbach’s ɑ = 0.89.

#### COVID-19- and pandemic-related questions

We asked participants several questions related to COVID-19 that were unrelated to the research question. They can be found in the materials as described above. These questions were followed by the German version of the Patient Health Questionnaire-4 (PHQ-4; Löwe et al., 2010), a questionnaire that served as a general screening for depression and anxiety with two items each. We adapted the scale by asking participants how they felt during the COVID-19-pandemic and, more specifically, how often they have encountered symptoms (e.g., “little interest or pleasure in your activities” for depression; “not being able to stop or control worries” for anxiety) on a four-point scale ranging from *not at all* to *almost every day*.

## Results

### Descriptive statistics and correlations

Descriptive statistics and bivariate correlations of all variables are presented in Table 3. Goal disengagement and goal reengagement were again positively correlated with each other, *r* = 0.39, 95% CI [0.21, 0.55], *t*(94) = 4.13, *p* < 0.001. Goal disengagement was not associated with measures of self-control or boredom, *p*s ≥ 0.13. In contrast, goal reengagement was positively correlated with both self-control, *r* = 0.21, 95% CI [0.01, 0.39], *t*(94) = 2.07, *p* = 0.041, and if–then planning, *r* = 0.41, 95% CI [0.23, 0.57], *t*(94) = 4.39, *p* < 0.001. Regarding boredom, goal reengagement was negatively correlated with boredom proneness, *r* = − 0.32, 95% CI [− 0.49, − 0.13], *t*(94) = 3.26, *p* = 0.002, but not significantly correlated with boredom avoidance and escape tendencies, *r* = − 0.03, 95% CI [− 0.23, 0.17], *t*(94) = 0.28, *p* = 0.778. As in Study 1, the strongest association emerged between self-control and boredom proneness.

**Table 3:** Means, standard deviations, and correlations of the main variables in Study 2

Variable	M	SD	1	2	3	4	5
1. Goal disengagement (GAS)	3.96	1.24					
2. Goal reengagement (GAS)	4.95	1.01	.39*** [.20, .55]				
3. Domain-general self-control (BSCS)	4.17	0.97	− .15 [− .34, .05]	.22* [.02, .40]			
4. If–then planning (ITPS)	5.02	0.76	.02 [− .18, .22]	.41*** [.23, .57]	.48*** [.31, .62]		
5. Boredom proneness (SBPS)	2.91	1.19	− .16 [− .34, .04]	− .32** [− .48, − .12]	− .62*** [− .73, − .48]	− .35*** [− .51, − .16]	
6. Boredom avoidance and escape (BAES)	3.66	1.35	− .10 [− .29, .11]	− .02 [− .22, .18]	.03 [− .18, .22]	.01 [− .19, .21]	.10 [− .10, .29]

Values in square brackets represent the 95% confidence interval (CI) of the correlation coefficient

*p < .05. **p < .01. ***p < .001

Going beyond Study 1, we also analyzed the relationship of anxiety during the COVID-19 pandemic and depression with goal adjustment, self-control, and boredom. Anxiety and depression correlated strongly with each other, *r* = 0.58, 95% CI [0.44, 0.70], *t*(95) = 7.02, *p* < 0.001, and as Table 4 depicts, with many of the main variables of our study. In particular, we observed small-to-moderate negative correlations of anxiety with goal disengagement, *r* = − 0.37, 95% CI [− 0.53, − 0.18], *t*(95) = 3.82, *p* < 0.001, and reengagement, *r* = − 0.24, 95% CI [− 0.42, − 0.04], *t*(95) = 2.40, *p* = 0.018. Similarly, depression was negatively correlated with goal disengagement, *r* = − 0.27, 95% CI [− 0.44, − 0.07], *t*(95) = 2.70 *p* = 0.008, and reengagement, *r* = − 0.38, 95% CI [− 0.54, − 0.20], *t*(95) = 4.05, *p* < 0.001. Anxiety and depression also correlated negatively with domain-general self-control and positively with boredom proneness, *p*s ≤ 0.002, and the correlations with boredom proneness were stronger. If–then planning was negatively associated with depression, *r* = − 0.35, 95% CI [− 0.51, − 0.16], *t*(95) = 3.59, *p* < 0.001, but not significantly so with anxiety, *r* = − 0.15, 95% CI [− 0.34, 0.06], *t*(95) = 1.44, *p* = 0.154, while neither of these variables was associated with boredom avoidance and escape tendencies, *p*s ≥ 0.81.

**Table 4:** Means and standard deviations of PHQ-4 scores, and correlations with the other main variables in Study 2

		Goal adjustment		Self-control		Boredom	
PHQ-4 subscale	*M* (SD)	Disengagement	Reengagement	Domain-general self-control	If–then planning	Boredom proneness	Boredom avoidance and escape
Anxiety	2.20 (0.79)	− .37*** [− 0.53, − 0.18]	− .24* [− 0.42, − 0.04]	− .31** [− 0.48, − 0.12]	− .15 [− 0.34, 0.06]	.43*** [0.25, 0.58]	− .02 [− 0.22, 0.18]
Depression	2.31 (0.71)	− .27** [− 0.44, − 0.07]	− .38*** [− 0.54, − 0.20]	− .39*** [− 0.55, − 0.21]	− .35*** [− 0.51, − 0.16]	.64*** [0.50, 0.74]	.02 [− 0.18, 0.22]

Values in square brackets represent the 95% confidence interval (CI) of the correlation coefficient

*p < .05. **p < .01. ***p < .001

### Regression analysis

As in Study 1, we regressed goal disengagement and reengagement on our measures of self-control and boredom (Table 5). Neither domain-general self-control nor if–then planning emerged as significant predictors of goal disengagement, *p*s ≥ 0.06. However, the effect of domain-general self-control became significant after adjusting for the boredom measures, which had no effects on goal disengagement themselves, *p*s ≥ 0.15: domain-general self-control then predicted lower goal disengagement, *b* = − 0.56, 95% CI [− 0.90, − 0.22], *t*(92) = 3.28, *p* = 0.001; β = − 0.44, 95% CI [− 0.70, − 0.17], and an effect in the same direction emerged for boredom proneness, *b* = − 0.40, 95% CI [− 0.66, − 0.14], *t*(92) = 3.09, *p* = 0.003; ꞵ = − 0.39, 95% CI [− 0.63, − 0.14].

**Table 5:** Regressing goal disengagement and goal reengagement on measures of self-control and boredom in Study 2

	Goal disengagement			Goal reengagement		
*N* = 97	M1	M2	M3	M1	M2	M3
Intercept	4.06*** (0.86)	4.68*** (0.46)	6.80*** (1.18)	2.16** (0.64)	5.70*** (0.36)	3.57*** (0.91)
Domain-general self-control	− 0.27 (0.15)		− 0.56** (0.17)	0.02 (0.11)		− 0.13 (0.13)
If–then planning	0.21 (0.19)		0.16 (0.18)	0.54*** (0.14)		0.51*** (0.14)
Boredom proneness		− 0.15 (0.11)	− 0.40** (0.13)		− 0.27** (0.08)	− 0.22* (0.10)
Boredom avoidance and escape		− 0.07 (0.09)	− 0.04 (0.09)		0.01 (0.07)	0.00 (0.07)
R^2^	.04	.03	.13	.17	.10	.21

The table shows unstandardized regression coefficients with standard errors in parentheses. *N* denotes the sample size

*p < .05. **p < .01. ***p < .001

Higher goal reengagement was predicted by if–then planning, *b* = 0.54, 95% CI [0.25, 0.82], *t*(94) = 3.75, *p* < 0.001; β = 0.40, 95% CI [0.19, 0.62], but not by domain-general self-control, *b* = 0.02, 95% CI [− 0.20, 0.24], *t*(94) = 0.22, *p* = 0.828; β = 0.02, 95% CI [− 0.19, 0.24]. Concerning boredom, boredom proneness predicted less reengagement, *b* = − 0.27, 95% CI [− 0.43, − 0.10], *t*(94) = 3.22, *p* = 0.002; ꞵ = − 0.32, 95% CI [− 0.51, − 0.12], while boredom avoidance and escape was no significant predictor, *b* < 0.01, 95% CI [− 0.14, 0.15], *t*(94) = 0.12, *p* = 0.907; β = 0.01, 95% CI [− 0.18, 0.21]. The effect of if–then planning, *b* = 0.51, 95% CI [0.23, 0.79], *t*(92) = 3.63, *p* < 0.001; β = 0.38, 95% CI [0.17, 0.60], and boredom proneness, *b* = − 0.22, 95% CI [− 0.42, − 0.02], *t*(92) = 2.19, *p* = 0.031; β = − 0.26, 95% CI [− 0.50, 0.02], remained significant in a model considering self-control and boredom jointly.

## Discussion

The results of Study 2 were largely consistent with our hypotheses. While goal reengagement was again robustly and positively associated with if–then planning, no association between goal disengagement and if–then planning was observed. This is consistent with research showing that if–then planning does not render goal striving tenacious when working toward the goal becomes unambiguously pointless (Legrand et al., 2017). Concerning domain-general self-control, we again found no robust association with goal reengagement. However, a negative association with goal disengagement emerged when adjusting for boredom. This was mirrored in the analysis of boredom proneness, pointing to the strong conceptual overlap between constructs, at least how they are currently conceptualized and measured: As people high in self-control tend to be low in boredom proneness (here: *r* = − 0.62), there are opposing effects on goal disengagement that cancel each other out unless the respective other construct is taken into account. Boredom proneness was also negatively associated with goal reengagement as in Study 1, whereas no association between reengagement and boredom avoidance and escape tendencies was observed. Together, these findings show that self-control and boredom have quite similar links to goal adjustment in general as well as to the adjustment of important personal goals in response to external barriers. The most apparent differences between Study 1 and 2 pertained to the negative effects of self-control and boredom proneness on goal disengagement, to which we turn in the general discussion.

## General discussion

In order to be successful in their goal striving, people have to effectively disengage from goals that become unattainable and reengage in meaningful alternative goals (Wrosch et al., 2003). In the present article, we have shown that individual differences in self-control and boredom are associated with differences in goal disengagement and reengagement. Across two studies, if–then planning had beneficial effects on goal reengagement, while goal disengagement was negatively associated with if–then planning in Study 1 and with domain-general self-control in Study 2. Goal reengagement was positively associated with boredom avoidance and escape tendencies in Study 1 and negatively associated with boredom proneness in Studies 1 and 2. As such, our results are consistent with theoretical propositions regarding the importance of individual differences in self-control and boredom for goal adjustment (e.g., Wolff & Martarelli, 2020; Bieleke & Wolff, 2021). They also highlight how essential it is to distinguish between goal disengagement and reengagement as distinct aspects of goal-adjustment, as they are differentially related to these personality factors (e.g., Wrosch & Scheier, 2020). For example, results across our two studies tended to be more robust with respect to goal reengagement than with regard to goal disengagement. This suggests that links between personality factors and goal reengagement might generalize more readily across different goals than links between personality and goal disengagement. Lending additional credit to this interpretation, we observed that personality factors explained more of the variance in goal reengagement than in goal disengagement. Finally, the results from Study 2 highlight the importance of goal adjustment, self-control, and boredom for mental health and well-being (e.g., Sommers & Vodanovich, 2000; Wrosch et al., 2003; Tangney et al., 2004) in dealing with unattainable goals during the COVID-19 pandemic.

## Limitations

The results of the present research make an important step toward a better understanding of the personality factors that contribute to goal adjustment; however, some limitations should be addressed as well. First, we were interested in the relationship between personality factors and therefore focused on cross-sectional data. Longitudinal assessments would be better suited for establishing the temporal order of the observed associations, thus providing a better approximation of causality (e.g., of the effects on mental health and well-being). Moreover, it would be interesting to scrutinize the nature of the interplay between self-control and boredom. Arguments have been put forward according to which either boredom mediates the associations between self-control and behavior (e.g., Boylan et al., 2021) or self-control moderates the association between boredom and behavior (e.g., Bieleke et al., 2021c). Understanding this interplay requires longitudinal or experimental data and might help elucidate their joint effects on goal adjustment. Second, we covered general tendencies of goal adjustment in Study 1 and then focused on the adjustment of an important personal goal in Study 2. While our two studies accordingly cover two essential aspects of goal adjustment, our findings relied on self-reported goal adjustment in both cases. This partly reflects that goal adjustment is an inherently subjective experience and not easily observable. Still, it would be interesting to see whether the associations between goal adjustment, self-control, and boredom also emerge when more objective measures of goal adjustment are used. Third, we focused on if–then planning as one specific self-control strategy that does not represent the full spectrum of self-control strategies people use in daily life (Duckworth et al., 2016; Hennecke et al., 2019). For instance, self-control strategies are often characterized as being based on either effortful or effortless processes (Gillebaart & de Ridder, 2015; Milyavskaya et al., 2019a). If–then planning, however, involves both effortful (e.g., thinking about when, where, and how to attain a goal) and effortless processes (e.g., automatically eliciting a goal-directed behavior; Webb & Sheeran, 2007) and might thus be considered a special case of a self-control strategy (e.g., Martiny-Huenger et al., 2016). Accordingly, future research should systematically investigate how effortful and effortless self-control strategies affect goal adjustment (e.g., Ainslie, 2021).

## Implications and future directions

Our findings have important implications for research on if–then planning, which has almost exclusively focused on the effects of making plans for a specific goal (e.g., as part of an intervention; Bieleke et al., 2021b). Our research suggests that the findings concerning the flexibility-rigidity tradeoff do only partly apply to individual differences in the inclination to make if–then plans in everyday life (Bieleke & Keller, 2021). On the one hand, we found a negative association between if–then planning and goal disengagement in Study 1, which disappeared when we focused on goal striving that was thwarted during the COVID-19 pandemic in Study 2. This is in line with the “flexible tenacity” commonly associated with the automating effects of if–then planning on behavior (e.g., Gollwitzer et al., 2008; Legrand et al., 2017). On the other hand, we also found reliable positive associations between if–then planning and goal reengagement across both studies. This might reflect that individuals who tend to make plans in their daily lives invest more effort into new goals, making these goals appear less difficult and more attainable. This observation sheds light on the complex ways in which if–then planning matters for goal adjustment and warrants future research investigating differences between if–then planning as an isolated intervention versus an individual difference variable.

Our results also provide novel evidence for the idea that boredom matters for goal adjustment and, in particular, for goal reengagement. The negative associations between boredom proneness and goal reengagement correspond well to the notion of boredom proneness as a “failure to launch” (Mugon et al., 2018). Importantly, however, an opposite pattern of association emerged between goal reengagement and boredom avoidance and escape tendencies in Study 1. This corroborates the proposition that boredom as a functional signal motivates explorative behavior (Danckert, 2019; Bieleke & Wolff, 2021). This is a crucial finding because it highlights the need to develop boredom measures that go beyond the current conceptualization of boredom proneness and that capture facets of trait boredom that have been neglected so far. Another intriguing finding pertains to the negative association between goal disengagement and boredom proneness in Study 2 that we did not observe in Study 1. This might be attributed to low levels of domain-general self-control among boredom-prone individuals (both in the present studies and in the literature; e.g., Wolff et al., 2022): In Study 1, we focused on general tendencies to disengage from unattainable goals in everyday life. Boredom-prone individuals might swiftly disengage from such goals when alternative activities require little self-control (e.g., watching Netflix) but struggle with disengagement when alternative activities require self-control (e.g., exercising; see Wolff et al., 2021), obfuscating the predicted association between boredom proneness and goal disengagement. In Study 2, in contrast, we focused on important goals that were frustrated by the COVID-19 pandemic. Given the limited set of activities during a pandemic (e.g., restriction of many routine activities), the available ones might have required more self-control (e.g., to obey government measures; Wolff et al., 2020), which might explain the negative association between boredom proneness and goal disengagement. This interpretation of our data seems worthwhile to be tested in future research.

Finally, one might argue that self-control and boredom represent but two examples of a potentially large number of personality factors involved in goal adjustment (e.g., Wrosch & Scheier, 2020; Brandstätter & Bernecker, 2022). Therefore, we think of the present research in terms of a promising starting point for more comprehensive analyses of the association between personality and goal adjustment that take a broader set of constructs into account. For instance, the tripartite model of goal striving (Ntoumanis & Sedikides, 2018) indicates that the effects of self-control strategies on goal adjustment might vary as a function of autonomous versus controlled motives for goal pursuit, and that this interaction is further shaped by a variety of personality factors like perfectionism and pessimism. Along similar lines, it seems promising to account for the degree of automaticity an individual has achieved with regard to the goal at hand. As if–then plans primarily operate via automating behavior, their effects on goal adjustment might be affected by differences in perceived habit strength (e.g., Verplanken & Orbell, 2003). Finally, it would be worthwhile to zoom into the specific goals participants pursue as a function of their tendency to engage in if–then planning or how boredom prone they are. For instance, people who rely on if–then planning might pursue more achievable goals as a result of thinking about potential obstacles; alternatively, their strategic preparation might encourage them to face more challenging goals. Importantly, differences in selected goals might explain the present findings (e.g., participants might be less likely to disengage from more achievable goals).

## Conclusion

Taken together, our findings shed light on self-control and boredom as two personality factors that are closely linked to effective disengagement from unattainable goals and reengagement in alternative goals. We found a rather complex but theoretically meaningful interplay of these constructs that highlights the importance of distinguishing between different facets of goal adjustment. For instance, if–then planning was positively and consistently associated with goal reengagement, whereas it was negatively associated with goal disengagement unless goal striving was frustrated by external circumstances. In turn, our findings show how research on goal adjustment can advance our knowledge about self-control and boredom. For example, measures of boredom proneness do not capture the motivation to explore the environment inherent to the experience of boredom, highlighting the importance of developing scales like the boredom avoidance and escape scale that tap better into these aspects.
